# Electroencephalographic Evidence for Individual Neural Inertia in Mice That Decreases With Time

**DOI:** 10.3389/fnsys.2021.787612

**Published:** 2022-01-14

**Authors:** Andrzej Z. Wasilczuk, Qing Cheng Meng, Andrew R. McKinstry-Wu

**Affiliations:** ^1^Department of Anesthesiology and Critical Care, University of Pennsylvania, Philadelphia, PA, United States; ^2^Department of Bioengineering, University of Pennsylvania, Philadelphia, PA, United States

**Keywords:** neural inertia, hysteresis, electroencephalography, machine learning, anesthesia, mice, isoflurane

## Abstract

Previous studies have demonstrated that the brain has an intrinsic resistance to changes in arousal state. This resistance is most easily measured at the population level in the setting of general anesthesia and has been termed neural inertia. To date, no study has attempted to determine neural inertia in individuals. We hypothesize that individuals with markedly increased or decreased neural inertia might be at increased risk for complications related to state transitions, from awareness under anesthesia, to delayed emergence or confusion/impairment after emergence. Hence, an improved theoretical and practical understanding of neural inertia may have the potential to identify individuals at increased risk for these complications. This study was designed to explicitly measure neural inertia in individuals and empirically test the stochastic model of neural inertia using spectral analysis of the murine EEG. EEG was measured after induction of and emergence from isoflurane administered near the EC_50_ dose for loss of righting in genetically inbred mice on a timescale that minimizes pharmacokinetic confounds. Neural inertia was assessed by employing classifiers constructed using linear discriminant or supervised machine learning methods to determine if features of EEG spectra reliably demonstrate path dependence at steady-state anesthesia. We also report the existence of neural inertia at the individual level, as well as the population level, and that neural inertia decreases over time, providing direct empirical evidence supporting the predictions of the stochastic model of neural inertia.

## Introduction

The factors determining whether an individual experiences complications during transitions between the awake and anesthetized states, including awareness under general anesthesia, delayed emergence, or delirium after anesthetic emergence, remain poorly understood. Individual variations in the functional barrier impeding entrance into and exit from the anesthetized state, that is defined as neural inertia, may contribute to risk for such complications (Warnaby et al., 2017). First described by Friedman et al. and characterized on the population level in both mice and flies (Friedman et al., 2010), electrophysiologic equivalents of neural inertia have also been described in intact rats and in slice models of cortical activity as well (Voss et al., 2012; Flores et al., 2017). Neural inertia produces a path dependence between induction into and emergence from the anesthetic state, which has been variably observed in humans using behavioral, fMRI, and EEG-derived methods (Warnaby et al., 2017; Kuizenga et al., 2018; Lewis et al., 2018; Ferreira et al., 2020; Huang et al., 2021). Neural inertia has been shown to be subject to genetic control (Joiner et al., 2013), making it a promising avenue for investigation into interindividual variation in anesthetic response. Despite this potential for individual differences in neural inertia contributing to variable anesthetic effects, many of the investigations of neural inertia have focused on the population level, leaving the question of interindividual variation incompletely explored. In spite of large interindividual variability relating to anesthetic sensitivity, the characterization of the inertial component of the anesthetic state has been observed to be conserved across individuals, uncoupled to species or anesthetic, when described at the behavioral level (McKinstry-Wu et al., 2019; Wasilczuk et al., 2020). One implication of neural inertia is that the neural activity from which behavior is derived displays hysteresis. As a consequence, drug concentration alone is insufficient to predict the current state. Therefore, it is critical to demonstrate and analyze neural inertia in the absence of pharmacokinetic effects. Such confounders have been the subject of some controversy (Colin et al., 2018; Proekt and Kelz, 2018, 2021; Sepúlveda et al., 2019; McKinstry-Wu et al., 2020); however, an experimental model employing extended steady state anesthesia may circumvent those complications. Near the population isoflurane EC_50_ for hypnosis—0.6% (McKinstry-Wu et al., 2019), we have observed that while individuals fluctuate between the awake and anesthetized state, a population-level behavioral steady state is reached within 2 h after anesthetic onset (McKinstry-Wu et al., 2019). By approaching the population EC_50_ either from a more anesthetized or awake state, we present a novel method to evaluate neural inertia that minimizes potential confounding effects of both pharmacokinetics and drug-concentration specific effects, as the only difference between induction and emergence will be the history of the initial condition, rather than the final drug concentration.

Proekt and Hudson (2018) have proposed a model that explains neural inertia as a consequence of random movement on an energy landscape, resulting in stochastic switching between awake and hypnotic states. This theoretical basis for neural inertia builds upon their earlier finding of spontaneous electrophysiologic switching state and the subsequent characterization of a parallel phenomenon of stochastic behavioral switching, both of which occur at anesthetic steady state (Hudson et al., 2014; McKinstry-Wu et al., 2019). The model describes anesthetic state switching as analogous to the Brownian motion of a particle in a two-well system, with the two wells being the awake and unconscious states, respectively. The relative depths of the wells determine how likely it is that the particle is in one or the other, and the frequency of transitions is determined by the noise of the system. Proekt and Hudson (2018) demonstrated mathematically, the proposed system produces neural inertia consistent with what has been previously observed (Joiner et al., 2013). The model also has clinical implications regarding desired (e.g., normal emergence) and undesired (e.g., intraoperative awareness) anesthetic state transitions. If state switching is stochastic, eliminating the rare instances of intraoperative awareness may prove nigh impossible with current EEG-based depth of anesthesia monitors. Similarly, delayed emergence cannot be predicted or treated with existing monitoring and pharmacologic tools. Importantly, Proekt and Hudson’s model also suggests new possible targets for intervention on those same problems. If the noise of the system can be modulated, the likelihood of a state transition can be altered, thereby better controlling when such transitions occur. Pharmacologically modulating system noise in fact appears to be an attainable goal, given evidence that different anesthetic agents produce different degrees of system noise (Wasilczuk et al., 2020). While Proekt and Hudson’s proposed model significantly informs a possible basis for neural inertia and suggests methods of system manipulation, it remains to be experimentally verified. One testable prediction of this model that remains to be explored is that with increased noise of the system or in the limit of time, neural inertia will dissipate.

Here, we examine whether individual neural inertia exists using an inbred population of C57BL/6J mice and an experimental model that examines spectral path dependence at an anesthetic steady state. By establishing individual neural inertia and its trend over time, we experimentally test the prediction made by the model proposed by Proekt and Hudson that neural inertia should collapse in the limit of time. Both goals have the potential to expand our understanding of neural inertia and open new avenues of investigation into individualized anesthetic delivery.

## Methods

### Animals

Studies were approved by the Institutional Animal Care and Use Committee at the University of Pennsylvania and were conducted in accordance with National Institutes of Health guidelines. Inbred C57BL/6J male mice (Jackson Laboratories, Bar Harbor, ME) aged 12–17 weeks were used for all studies (*n* = 26, total).

### Isoflurane Exposure for Brain Drug Concentration Measurement

Individual animals received one of two isoflurane exposure paradigms. Animals in the “wash-in arm” (induction) received 0.9% isoflurane in 100% oxygen in air-tight 200 ml cylindrical chambers at 200 ml/min (*n* = 12). Animals in the “wash-out arm” (emergence) received 30 min of 0.9% isoflurane in 100% oxygen, followed by 100% oxygen alone (*n* = 8) in identical chambers. Mice were kept normothermic by having chambers be partially submerged in a temperature-controlled water bath (Sun et al., 2006). Isoflurane concentrations were confirmed using a Riken FI-21 refractometer (AM Bickford, NY). At the change in concentration to 0.9% (induction) or 100% oxygen (emergence) and various time points after, mice were rapidly sacrificed by cervical dislocation in order to obtain pharmacokinetic wash in and wash out equilibration curves. The mean time from the mouse being removed from the anesthetic chamber to cervical dislocation was 4.6 ± 1.8 s (mean ± std). The mean time from mouse being removed from the anesthetic chamber to the brain being flash-frozen in liquid nitrogen was 36.2 ± 5.3 s. The mean core trunk temperature for mice at the time of brain harvesting was 36.9 ± 0.5°C. Brains were subjected to same-day HPLC analysis as described below to determine brain isoflurane concentration.

### Direct Quantification of Whole-Brain Isoflurane Concentration

#### Preparation of Standards

Ten micromolar of isoflurane was prepared in methanol, and 5, 10, 15, 20, 25 μl of the isoflurane solution were injected into the HPLC to prepare the calibration curve, respectively. Calibration curves were constructed by plotting the height of the isoflurane peak against the known amount of analytes and fitted using linear regression analysis (Kelz et al., 2008).

#### Brain Sample Preparation

Frozen brain tissue was placed into 1 ml of mixture solution (acetonitrile:H_2_O 2:1). After homogenizing, the suspensions were centrifuged at 4°C at 20,000 *g* for 20 min. Fifty microliter of the resulting supernatant was injected into the HPLC.

#### HPLC Conditions

The HPLC system consisted of gold 126 solvent modules (Beckman Coulter), a RID-10A differential refractometric detector (Shimadzu, Kyoto, Japan), an analytical C_18_ column (Zorbax 300SB-C_18_ 250 mm × 4.6 mm I.D., 5 μm particle size; Agilent Technologies), and a one-channel recorder (Klipp and Zonen BD 40, Rotterdam, The Netherlands). The mobile phase, a mixture of acetonitrile, isopropanol, 0.02 M phosphate buffer, pH = 4.6 (340:150:510, v/v) was eluted at 1.0 ml/min.

#### Accuracy and Precisions

Chromatographic peaks for isoflurane were identified by retention times from the standard solution. Isoflurane was assayed by measuring the chromatographic height (mAu), and the amount determined from the standard calibration curve that was prepared daily. Drug-free brain samples were used in the generation of calibration, negative control, and quality control.

### Isoflurane Wash-In and Wash-Out Curves

Curve fitting and goodness-of-fit calculations were performed using Prism 9.2 (Graphpad Software Inc., San Diego, CA). Kinetics were modeled using exponential functions, wash-in with exponential plateau, while wash-out was modeled using exponential one-phase decay.

### Chronic EEG Implantation

Mice (*n* = 6) were chronically implanted with 25 lead EEG and four EMG, as described previously (Wasilczuk et al., 2016). In brief, mice underwent anesthetic induction with 2.5% isoflurane in 100% oxygen and were maintained on 1.5% isoflurane for the surgery. After confirming nonresponsiveness to a toe pinch, mice were placed on a stereotaxic frame (Kopf Model 902, Kopf Instruments, Tujunga, CA), eye ointment applied, and core temperature was maintained at 37 ± 0.5°C using a closed-loop servo-controlled heating pad (CWE Inc, Ardmore PA). A total of 26 epidural EEG leads were implanted (25 plus ground lead), across both halves of the skull at 1.00 mm and 2.30 mm lateral to the midline. Implanted leads were spaced 3.30 mm anterior to 4.50 mm posterior to Bregma in 1.30 mm increments. For the more lateral leads, contacts were arrayed from 2.00 mm anterior to Bregma to 4.50 mm posterior to Bregma with 1.30 mm between contacts. Additionally, two EMG leads were implanted in the dorsal neck muscles, and two EMG leads in the upper back. The headpiece was secured to the skull using dental cement (A.M. Systems, Carolsburg WA). All mice were allowed to recover for a minimum of 2 weeks prior to drug exposure and EEG/EMG recordings.

### Isoflurane Exposure for EEG Acquisition

**Figure 1:**
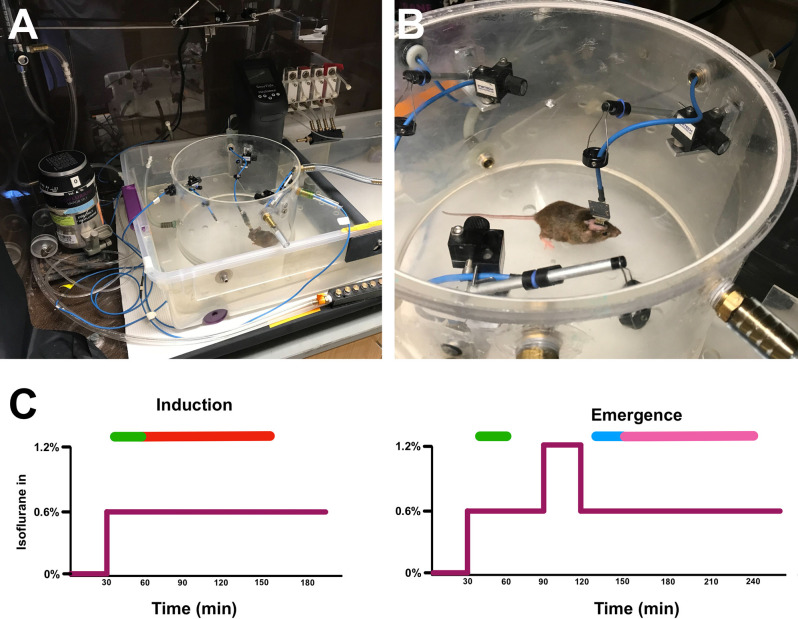
Experimental setup and isoflurane exposure paradigm. Tethered mouse in open **(A)** and sealed **(B)** cylindrical recording chambers in water bath. **(C)** The induction (left) and emergence (right) isoflurane exposure paradigms.The purple line represents isoflurane concentration over time. Green bars represent time periods used for induction classifier training, the blue bar is the time period used for emergence classifier training, the red bar is the time period of induction on which classifiers were tested, and the magenta bar is the time period of emergence on which classifiers were tested (See “Classifier Construction and Analysis” section in Methods).

Upon surgical recovery, mice were habituated to being tethered to the headstage amplifier within the recording area for an hour a day for three consecutive days. On recording days, mice were attached to headstage amplifiers, connected to the EEG acquisition system (described below), and placed inside 8 L gas-tight cylindrical recording chambers. Gas flow through the chambers was set at 8 L/min. The chambers were maintained at 37°C *via* partial submersion in a circulating water bath, keeping mice normothermic under the anesthetic exposure. Each EEG-implanted mouse received two separate isoflurane exposures, separated by at least 7 days. In the induction paradigm, mice received a 100% oxygen exposure for 30 min followed by 0.6% isofluranein 100% oxygen for 240 min. In the emergence paradigm, mice received a 30-min 100% oxygen baseline, 60 min of 0.6% isoflurane, 30 min of 1.2% isoflurane, and finally 0.6% isoflurane for 240 min (Figure 1).

### EEG Acquisition and Preprocessing

**Figure 2:**
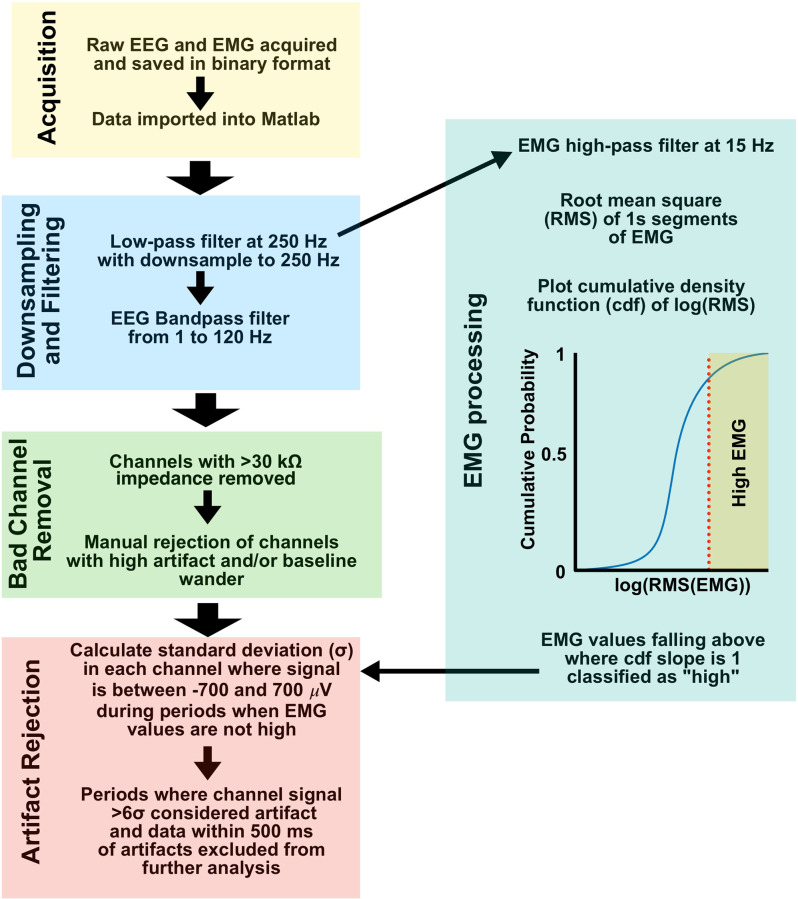
EEG and EMG signal preprocessing. Workflow from acquisition to artifact rejection was used for all data in this study. EEG, electroencephalogram; EMG, Electromyography.

All EEG was recorded using an acquisition system constructed according to open source designs available from Open-Ephys (Siegle et al., 2017), 32-channel headstage amplifiers (RHD 2132, Intan Technologies, Los Angeles, CA), and the Open-Ephys GUI software v2.0^1^. Recordings were imported into Matlab 2021b (Mathworks, Natick MA) for post-processing. Figure 2 shows a summary of signal preprocessing. All signals were low-pass filtered and downsampled to 250 Hz from their original acquisition rate of 1 kHz using the Matlab *decimate* function. EEG was bandpass filtered between 1 and 120 Hz using a 6th order zero-phase Butterworth filter prior to additional processing. Impedance measurements were taken prior to recordings and leads with an impedance measurement greater than 30 kΩ were excluded from the analysis. Channels were further manually inspected, and channels with excessive artifact or baseline wander were excluded from further analysis. To identify artifacts, the standard deviation (*σ)* of the signal voltage from each channel was taken, excluding time periods where the signal exceeded ±700 μV and during periods of exceptionally high EMG tone where the signal could be contaminated with EMG artifact (see below). Any period from the entire channel where the signal exceeded the calculated 6*σ* was labeled as artifactual, and that channel-period was excluded from further analysis. Channels were mean re-referenced once artifact detection excluded erroneous signals from the recordings.

### EMG Processing and High EMG/Movement Classification

EMG activity and movement as defined by 3-axis accelerometry have been shown to be strongly correlated in mice, leading to the use of EMG activity as an artifact rejection routine for our EEG preprocessing (Wasilczuk et al., 2018). The single EMG lead most free of cardiac and other visible artifacts was chosen for analysis for each subject. EMG was low-pass filtered and downsampled to 250 Hz using the Matlab *decimate* function, and high-pass filtered at 15 Hz using a 6th order zero-phase Butterworth filter. The root mean squared (RMS) value for 1 s non-overlapping periods was calculated, and a histogram of the cumulative density function estimate for the log RMS value was plotted. On the resulting sigmoid plot, RMS EMG values greater than where the slope of the cumulative density function estimate curve drops below 1 were considered “high” EMG tone, and a potential source of movement artifact in EEG (see above and Figure 2).

### Spectral Analysis and Dimensionality Reduction

**Figure 3:**
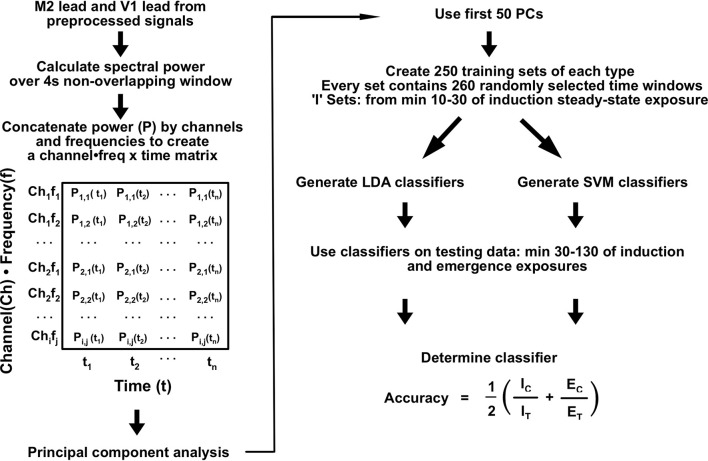
EEG analysis process summary.

Signals from the secondary motor cortex (M2; 2.0 mm anterior and 1.0 mm lateral to Bregma) and primary visual cortex (V1; 4.5 mm posterior and 2.30 mm lateral to Bregma) were used for all subsequently described analyses. Spectral power estimation was computed over 4 s non-overlapping windows using previously published code (Hudson et al., 2014). For dimensionality reduction, spectral power in each individual for the M2 and V1 channels was concatenated to a 482-dimensional vector (241 independent frequency estimates per channel) for each spectral window (Figure 3). The spectrum for each channel was expressed as differences from the mean spectrum determined over the entirety of the induction, emergence, and induction arm of the emergence recordings. The resulting matrix was subjected to principal component analysis (PCA). The first 50 principal components (PCs), representing >70% of the variance for each animal, were used for linear and nonlinear classifier construction and analysis.

### Classifier Construction and Analysis

Training sets were compiled using the first 50 PCs of spectral data from M2 and V1 for each individual mouse. Training sets consisted of equal amounts of 260 randomly-sampled windows from minutes 10–30 of the emergence arm and from minutes 10–30 for the induction arms of both exposures that each mouse received (Figure 1C). Testing sets were composed of the same features for minutes 30–130 after the start of the final 0.6% isoflurane administration for both the induction and emergence arms. Two-hundred and fifty classifiers were constructed through linear discriminant analysis (LDA) for each animal using the *fitclinear* Matlab function. Using the same training data, 250 support vector machine (SVM) classifiers were also generated using the *fitcsvm* Matlab function. For training on population-level data, training sets were generated from 1,300 spectral windows from minutes 10–30 for the emergence arms and from min 10–30 for the induction arms equally drawn from the five mice other than the mouse for which the classifier was tested on (e.g., data from mice 1, 3, 4, 5, and 6 were used to train the classifier for mouse 2). The accuracy of each classifier was calculated as


$$Classifier\;Accuracy=\frac{1}{2}\left(\frac{I_{C}}{I_{T}}+\frac{E_{C}}{E_{T}}\right)$$

where I_C_ is the number of correct induction labels, I_T_ is the total number of induction timepoints, E_C_ is the number of correct emergence labels, and E_T_ is the total number of emergence timepoints. A summary of the analysis process is shown in Figure 3.

### Quantification and Statistical Analysis

Data were analyzed using MATLAB 2021b using the Statistics and Machine Learning Toolbox and the Signal Processing Toolbox and Prism 9.2. All data were tested for normality, and non-parametric statistical tests wereused where data were found to be non-normal. A p-value < 0.05 was considered statistically significant for all comparisons. Indications of significance are as follows: * *p* <0.05; ** *p* < 0.01; *** *p* < 0.001; **** *p* < 0.0001.

## Results

### Isoflurane Brain Concentration Steady State

**Figure 4:**
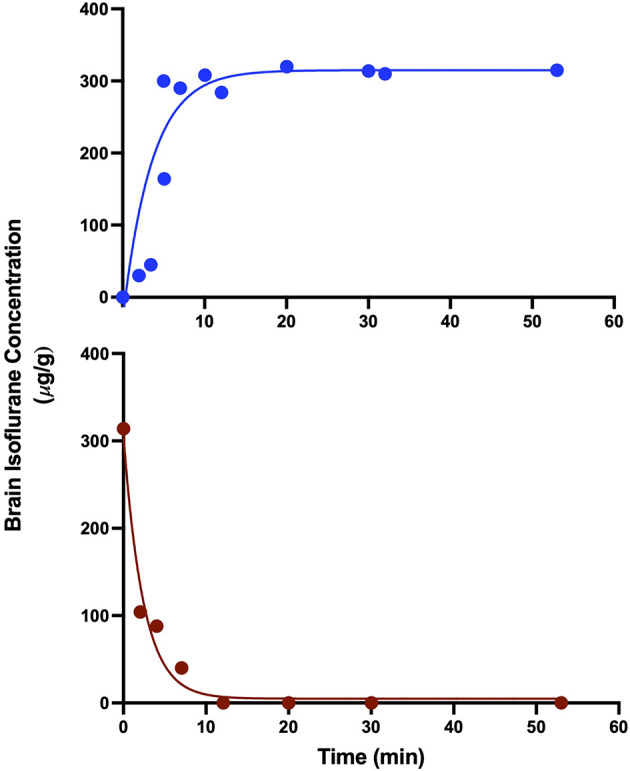
Brain isoflurane concentration reaches steady state in 8–12 min. Mice starting at 100% O_2_ and exposed to 0.9% isoflurane (top) reach steady state by 8 min, while mice exposed to 0.9% isoflurane for 30 min, followed by 100% O_2_ (bottom) reach undetectable levels of isoflurane in the brain by 12 min after the start of O_2_ administration. R^2^ values for exponential fits were 0.784 for wash-in and 0.974 for washout.

In order to determine an estimated time course for isoflurane anesthetic steady-state in the brain, individual mice received a wash-in or wash-out exposure of 0.9% isoflurane for varying lengths of time, and whole brain anesthetic concentration was determined using HPLC. With gas delivery to the sealed chambers at 1 volume turnover every minute, brain concentrations rapidly reach steady state equilibration in the wash-in (induction) and wash-out (emergence) paradigm in under 12 min (Figure 4). Since this pharmacokinetic estimate was performed at the population level, in order to go far beyond any possibility variation by any individual, we chose to perform subsequent analyses on data acquired more than twice as far out, 30 min after concentration changes and later.

### Differences in Mean EEG Spectra and Dynamic Spectra Between Induction and Emergence

**Figure 5:**
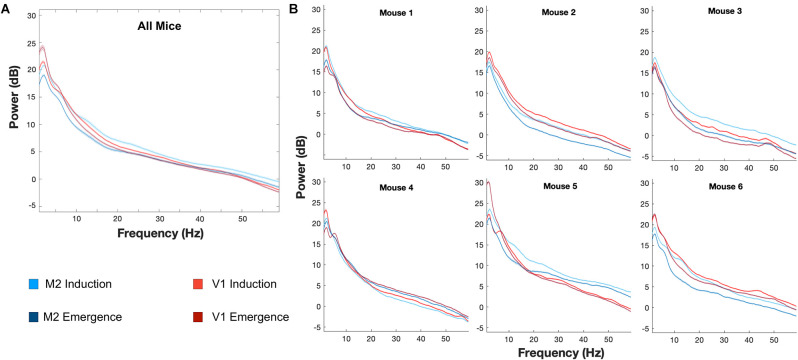
Individual differences in induction and emergence evident in mean spectrum. Mean spectra across animals at M2 and V1 **(A)** demonstrate significant differences between induction and emergence. The mean spectra, however, mask the notable variation of spectra, and various differences between induction and emergence spectra, that occur in individual animals **(B)**. Lines represent the mean spectrum and shaded areas indicate the 99.979% confidence interval of the mean, corresponding to a *p* < 0.05 with Bonferroni correction for the multiple comparisons corresponding to each frequency. M2: Blue, V1: Red, Induction: Lighter Shade, Emergence: Darker Shade.

**Figure 6:**
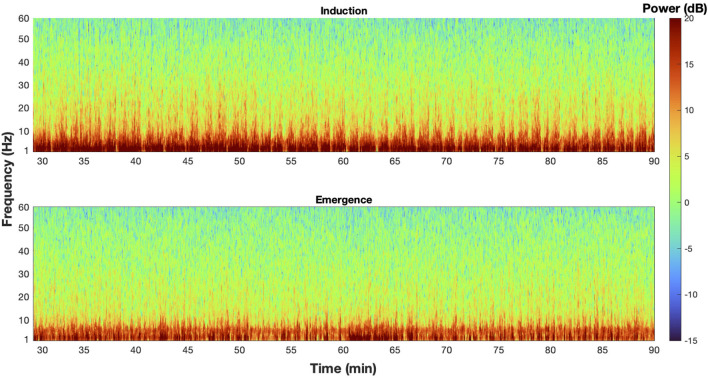
The EEG spectra at steady state is dynamic. An example spectrogram from M2 of mouse 1 during induction (top) and emergence (bottom) exposures that illustrate the dynamic nature of the spectra at steady state, and how taking a mean spectrum erases potentially critical information from the signal.

Mice chronically implanted with EEG and EMG each underwent two exposure paradigms, separated by at least a week. Analysis of EEG began at least 30 min after the switch to the final 0.6% isoflurane concentrations. Given that brain isoflurane levels reached steady-state within 12 min (Figure 4), we minimized potential pharmacokinetic confounding by limiting our analysis to a 100-min window that started 30 min into the final concentration step (minutes 30–130). For each animal, at the two common leads (over M2 and V1), there were significant differences between mean spectra after induction and emergence (Figure 5A). Such averaging across the population, however, masks the substantial variation seen among individuals (Figure 5B). Nevertheless, examined either way there is a significant spectral difference in the period from 30 to 130 min after concentration change to 0.6% between a history of moving from a lower versus a higher concentration. EEG at anesthetic steady state is not static (Hudson et al., 2014; Shortal et al., 2019), however, and just as averaging across the population masks spectral differences among individuals, taking the mean spectrum eliminates the not inconsiderable dynamics present in the signal (Figure 6). The dynamic discrete fluctuations evident in the spectrogram are not unlike the behavioral fluctuations seen at hypnotic EC_50_, and like those fluctuations, averaging them over time can obscure potentially important information (McKinstry-Wu et al., 2019; Wasilczuk et al., 2020).

### Differentiability of Induction and Emergence EEG Spectra: Neural Inertia in Individuals

An accurate quantification of the difference between induction and emergence in the EEG spectra must be able to account for interindividual spectral variation as well as spectral dynamics. Towards this, we employed linear discriminant analysis (LDA) as well as a supervised machine-learning algorithm, support vector machine (SVM), to develop classifiers on the first 50 principal components of the concatenated channel-frequency power spectrum from our two common channels (over M2 and V1). Together, these first 50 principal components account for 70–78% of the variance of the original data for each animal, while reducing the dimensions of the data by nearly a factor of 10. Classifiers (250 per animal) were trained on data from randomly selected times from 10–30 min after the concentration switch to 0.6% isoflurane. In order to account for any potential differences between recordings in the same animal, induction training data were drawn equally from both the induction paradigm as well as the first 0.6% step of the emergence paradigm (the induction arm of the emergence paradigm recording).

**Figure 7:**
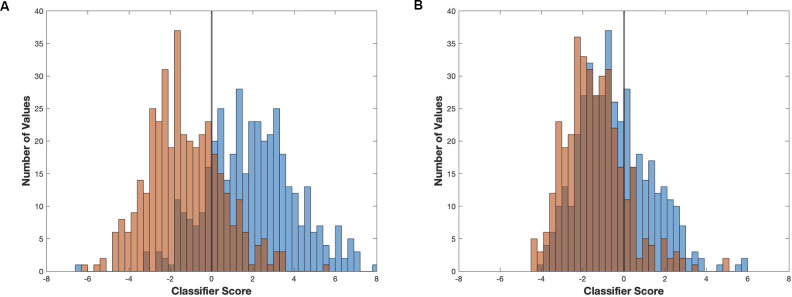
Linear classifier accuracy determines maximum separability of two signals. Two examples of linear classifiers demonstrating how classifier differentiation can vary in effectiveness at separating two signals. Examples using linear classifier models and resulting scores generated from **(A)** the first 2,000 s of induction (blue) and emergence (red) from mouse 2 (higher accuracy/effectiveness,) and **(B)** the final 2,000 s of induction (blue) and emergence (red) from mouse 1 (low/chance accuracy/effectiveness).

**Figure 8:**
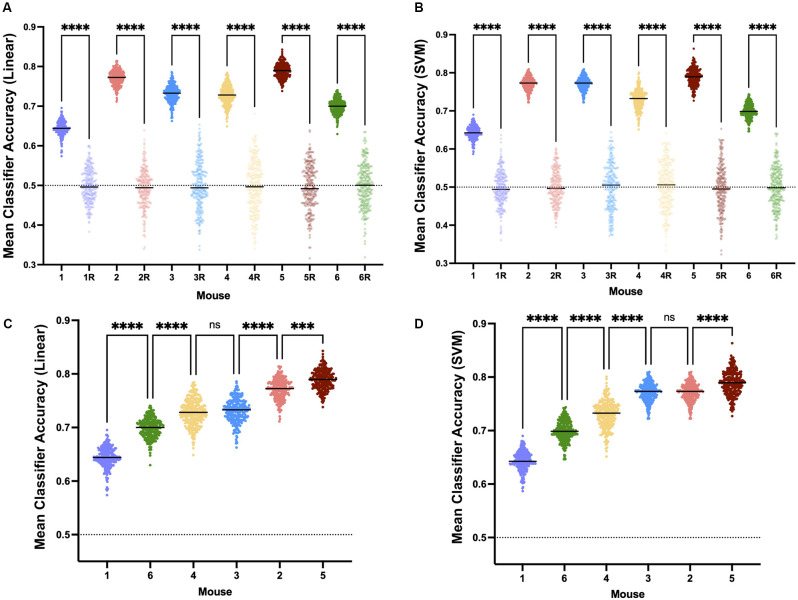
Neural inertia is present in individuals. Individuals show significant neural inertia over the 100 min beginning 30 min after the final 0.6% isoflurane step, as measured by linear classifier accuracy indicating quantifiable distinctions in spectra based on whether the mouse was previously awake or deeply anesthetized. Points represent the mean accuracy over the measured period for a given classifier. Both linear classifiers **(A)** and support vector machine generated classifiers **(B)** correctly were able to distinguish induction vs. emergence based on spectral distinctions at a level greater than chance and significantly (*p* < 0.0001) more than classifiers using shuffled training data (randomly shuffled data indicated with “R”.) Relative classifier accuracy and differences between individuals were largely conserved across approaches (**C** and **D**, *p* = 0.4896, Friedman test), suggesting interindividual variability in neural inertia. All comparisons made using Kruskal-Wallis test. ns, not significant, ***p < 0.01, ****p < 0.001.

**Figure 9:**
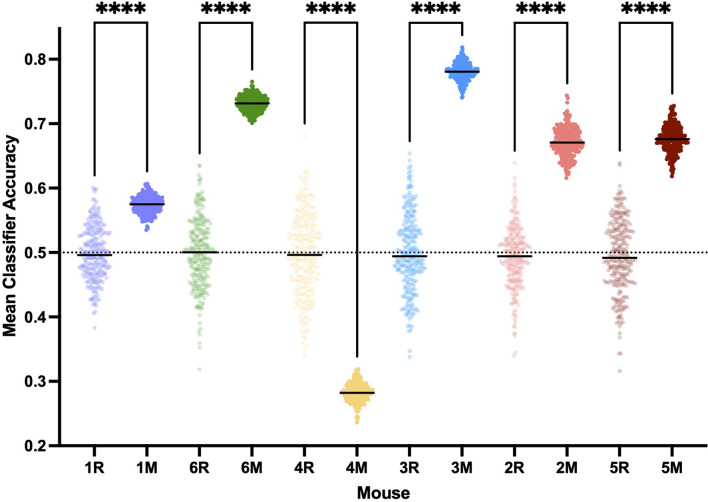
Classifying bypopulation spectra detects individual neural inertia less efficientlythan individualized classification. Training linear classifiers onpopulation data resulted in classifier accuracy greater than chance(*p* < 0.0001, Kruskal-Wallis) and thus evidence for neuralinertia in five of the six mice, rather than detecting neural inertia in all mice 6 with individualized training sets. ****p < 0.001.

A linear classifier identifies an axis of maximum distinction between two data sets and will effectively account for interindividual differences. SVM can construct nonlinear binary classification on similar datasets. If the induction and emergence spectra were sufficiently distinct (high accuracy, Figure 7A), a classifier would have a significantly greater than a chance probability of determining if the spectrum in question was drawn from an induction or emergence exposure paradigm. Hence, successful classification of an EEG spectrum attributable to either induction or emergence would be proof of path dependence that defines neural inertia. If neural inertia is not present or has collapsed, there should be little difference between the induction and emergence EEG spectral signals. In this latter case, we would expect the classifier to do a poor job (chance or equivalent) of distinguishing channel-spectra of the induction exposure from channel-spectra of the emergence exposure (low accuracy, Figure 7B). While shuffled training data produced classifiers that were no better than chance (95% confidence intervals of the median overlap 0.5), both the linear and SVM classifiers accurately assigned the real spectral data significantly greater than chance (*p* < 0.0001) in all mice. This is concrete evidence that all mice displayed significant levels of neural inertia over the 100 min beginning 30 min after the start of the final 0.6% isoflurane step (Figures 8A,B). We were not able to detect any differences between LDA and SVM classifier accuracy (*p* > 0.05, Friedman test). Both methods of classification showed similar and significant differences between mice in neural inertia, suggesting that such interindividual differences may be robust (Figures 8C,D). Population based classification, using training data from five mice to classify the sixth, was not as effective as individualized training. Nevertheless, it demonstrated neural inertia in five of six of the mice (Figure 9). Thus, while population data proved an effective means of detecting neural inertia in most cases, individualized methods were able to detect spectral differences where population-level methods could not.

### Change in Neural Inertia Over Time

**Figure 10:**
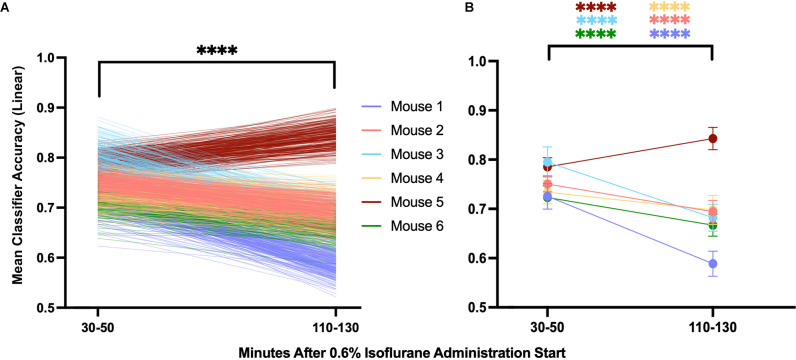
Population and individual neural inertia decrease over time. Neural inertia as measured by mean classifier accuracy was compared over the first 20 and final 20 min of the 100 min beginning 30 min after the final 0.6% isoflurane step. Across animals **(A)**, neural inertia showed a significant decrease over the measured period (values from individuals shown by color). By individual **(B)**, five of six animals showed a significant decrease in neural inertia, while the outlier (mouse 5) had an increase in neural inertia over the same period (Wilcoxon matched-pairs signed rank test and two-way ANOVA with Sídák’s multiple comparisons test, respectively). ****p < 0.001.

If the model proposed by Proekt and Hudson (2018), describing induction and emergence in terms of Brownian Motion over a two-well energy landscape, is fundamentally correct, then it follows that the degree of neural inertia, i.e., the linear classifier accuracy, should decrease to chance (50%). To examine this trend over time, we evaluated the mean classifier accuracy over the first 20 min of our analysis (minutes 30–50 after beginning 0.6% isoflurane) and compared that to the last 20 min (minutes 110–130 after beginning 0.6% isoflurane). We found a significant decrease in the accuracy of the classifier between the two periods (Figure 10A, *p* < 0.0001), consistent perhaps with a decrease in neural inertia. Individually, five of six of the mice showed a significant decrease in classifier performance over the examined period, with the outlier showing a significant increase over the same period (Figure 10B, *p* < 0.0001). These differences are not artifacts of the model, as shuffled training data shows no change over the same period (Supplementary Figure 1). Taken together, this evidence for neural inertia decreasing over time provides confirmatory experimental support for the Proekt-Hudson stochastic model of neural inertia (Proekt and Hudson, 2018).

## Discussion

Using a novel approach at steady-state population EC_50_ for isoflurane, we show that differences in EEG spectra solely dependent on a history of awake or deeply anesthetized states, consistent with neural inertia, not only occur but are present on a timescale unrelated to potential confounding effects of pharmacokinetics. Moreover, evidence for neural inertia can be seen at the individual level. Finally, we demonstrate that classifier efficacy decreases over time, consistent with a decrease in neural inertia, providing the first experimental evidence in support of the stochastic model of neural inertia proposed by Proekt and Hudson.

While the extent of neural inertia seen in individuals may indeed correlate to a larger barrier to state transitions, there are assumptions that must be made for that conclusion to be valid. One such assumption is that the amount of noise across recordings is constant. Differential amounts of noise directly would impact the resolving power of spectral window classification as a function of the initial state, and noise can be introduced in a variety of ways into a recording. We mitigated this possibility, however, through the examination of impedance measurements of EEG and EMG leads across recordings, and meticulous preprocessing of the EEG. Similarly, neural inertia as computed here relies on classification techniques that could result in different conclusions based on the underlying structure of the input data. This seems less likely given that both linear and nonlinear machine learning methods reliably distinguished spectral windows between induction and emergence paradigms. The robustness of both classifiers suggests that neural inertia exists, and this conclusion is strengthened by the lack of significant differences in accuracy by classifier choice. The suggestion of variability in individual neural inertia, indicated by persistent interindividual differences between classifiers, begs the question of the relationship between neural inertia and another measure of behavioral anesthetic response we previously described, resistance to state transition (RST; Wasilczuk et al., 2020). The intuitive nature of the relationship between RST and neural inertia and the one explicitly predicted by the stochastic model of neural inertia, is that neural inertia is directly predicted from RST. Such a relationship is further supported by behavioral evidence of increased neural inertia with agents that produce a higher RST, such as with halothane. We do not purport to measure an EEG equivalent of RST here, which would require discretizing the EEG signal into two or more states reproducibly across animals, but instead are examining a continuous variable measuring a net difference between two signals that are analogous to the path dependence observed between population-level induction and emergence curves, neural inertia.RST has only been measured at the *behavioral* steady state, whereas we are measuring neural inertia during a period of only *pharmacologic* steady state, where behavior may still be changing during this time.Furthermore, RST will still exist even after neural inertia collapses due to the persistence of the dynamics of the system, emphasizing that neural inertia is a consequence of a system with RST.

The direct relationship between RST and neural inertiapredicted by the stochastic model makes the discrepancy between the relatively low amount of inter individual variability seen in RST and the high variability of neural inertia we observe here all the more surprising (Wasilczuk et al., 2020), particularly as it occurs all within the same ostensibly homogenous population of C57BL/6J mice. One potential explanation for the difference in variability between righting-reflex-based RST and EEG-derived neural inertia is the qualitative difference between the two measures. Righting reflex measurements produce binary outcomesin response to stimuli, while cortical activity measured by EEG is nonlinear, non-binary, and a passive measure. Recent reports have attempted to correlate behavioral measures with EEG measures of anesthetic assessment, in both mice and humans, and have found that behavioral measures unreliably correlate to measures of EEG with respect to anesthetic state transitions (Haberham et al., 1999; MacIver and Bland, 2014; Shortal et al., 2019; Gao and Calderon, 2020). Future studies using the methods here in conjunction with other volatile anesthetics producing differing RSTs could serve to better explore the exact relationship between RST and neural inertia.

How other volatile agents affect individual neural inertia and inertial variability remains unknown. Whether relative neural inertia is conserved across anesthetic agents is similarly unknown. An investigation of neural inertia using different volatile anesthetic agents would have the added benefit of testing the other prediction of the model of neural inertia proposed by (Proekt and Hudson, 2018). While our data suggest that neural inertia decreases as time progresses, the stochastic model also predicts that neural inertia should collapse with increasing noise of the system. We previously estimated different amounts of noise driving behavioral state transitions dependent on volatile agent (Wasilczuk et al., 2020). According to the stochastic model of neural inertia, an individual’s level of neural inertia with isoflurane should be lower than that same individual’s neural inertia with halothane, which produces a lower level of system noise than isoflurane. This leads to a testable prediction, in the context of the analyses described, that neural inertia (i.e., classifier discrimination accuracy between induction and emergence spectral windows) would be greater in the same mouse when exposed to halothane than when exposed to isoflurane.

While there was a decrease in neural inertia across the population over the 100-min period examined, the degree of decrease was not consistent across individuals, which could be due to a number of factors. If there were significant inherent variability between recordings in the same animal, the classifier would discriminate between the two signals based on those differences rather than differences stemming from an induction or emergence exposure. Inherent recording differences such as those would not decrease over time, blunting or eliminating any decrease in neural inertia. We attempted to control for such inherent variability between EEG recordings by both mean re-referencing and training our classifiers on induction exposure data from two separate recording days. Another possibility is that neural inertia could rapidly collapse in some individuals, as the Proekt-Hudson model does not predict anything about the time scale on which neural inertia collapses, other than that the collapse should be independent of pharmacokinetic confounders (Proekt and Hudson, 2018). While we based our upper estimate of the time scale of neural inertia collapse on when we see a behaviorally-derived steady state (McKinstry-Wu et al., 2019; Wasilczuk et al., 2020), this may also not be a safe assumption, and neural inertia could collapse on a timescale longer than several hours. The time course of collapse may additionally vary by species, and may have a role in the variable detection of neural inertia in human studies to date (Warnaby et al., 2017; Colin et al., 2018; Kuizenga et al., 2018; Ferreira et al., 2020; Huang et al., 2021).

It is important that we acknowledge we use a measure of net neural activity, EEG spectra, to define neural inertia rather than a behavioral measure, which is how neural inertia was first defined. While true that all behavior is ultimately derived as a consequence of neural activity, measures of net neural activity and arousal behavior can and have been shown to diverge (Pal et al., 2020). Future studies can and should focus on examining behavioral correlates to the phenomena described here. Ideally, such studies will include both measures of EEG as well as behavior, so that we might better understand the relationship between spontaneous measures of neural activity and arousal behavior.

The degree of individual variation in the classifier performance was surprising and unexpectedly paralleled our earlier finding of significant individual variability in anesthetic sensitivity visible at population EC_50_ (McKinstry-Wu et al., 2019). These two previously unrecognized features of variability in individual anesthetic responsiveness suggest possible sources of risk for disorders of anesthetic transition. An individual with a low anesthetic sensitivity and low neural inertia might be predisposed to awareness under general anesthesia, while one with high sensitivity and high inertia could be predisposed to delayed emergence. Such possibilities suggest the need to explore human correlates of spectrally evident individual inertia at steady state. The steady state anesthetic model employed here could be modified and adapted for use in human studies, which may avoid some of the controversies surrounding pharmacokinetic confounds of measuring neural inertia (Colin et al., 2018; Proekt and Kelz, 2018, 2021; Sepúlveda et al., 2019; McKinstry-Wu et al., 2020).

## Data Availability Statement

The raw data supporting the conclusions of this article will be made available by the authors, without undue reservation.

## Ethics Statement

The animal study was reviewed and approved by University of Pennsylvania Institutional Animal Care and Use Committee.

## Author Contributions

AW and AM-W: conceptualization and funding acquisition. AW, QM, and AM-W: data curation, writing—review and editing. AM-W: formal analysis and writing—original draft. All authors contributed to the article and approved the submitted version.

## Conflict of Interest

The authors declare that the research was conducted in the absence of any commercial or financial relationships that could be construed as a potential conflict of interest.

## Publisher’s Note

All claims expressed in this article are solely those of the authors and do not necessarily represent those of their affiliated organizations, or those of the publisher, the editors and the reviewers. Any product that may be evaluated in this article, or claim that may be made by its manufacturer, is not guaranteed or endorsed by the publisher.

## Footnotes

^1^ www.open-ephys.org

## Supplementary Materials

Supplementary Figure 1 Classifier accuracy built on shuffled training data does not trend from chance over time. The accuracy of linear classifiers built from shuffled training data do not trend in any direction over time (*p* = 0.4288, 2-way ANOVA), but rather remain at the chance. This demonstrates a lack of inherent directional bias of the model itself over time, suggesting trends over time seen with non-shuffled training data are real.
