# Supplementary figures and images for: Electroencephalographic Evidence for Individual Neural Inertia in Mice That Decreases With Time
Source: Front Syst Neurosci. 2022 Jan 14;15:787612. doi: 10.3389/fnsys.2021.787612 (PMC8794956; doi:10.3389/fnsys.2021.787612)

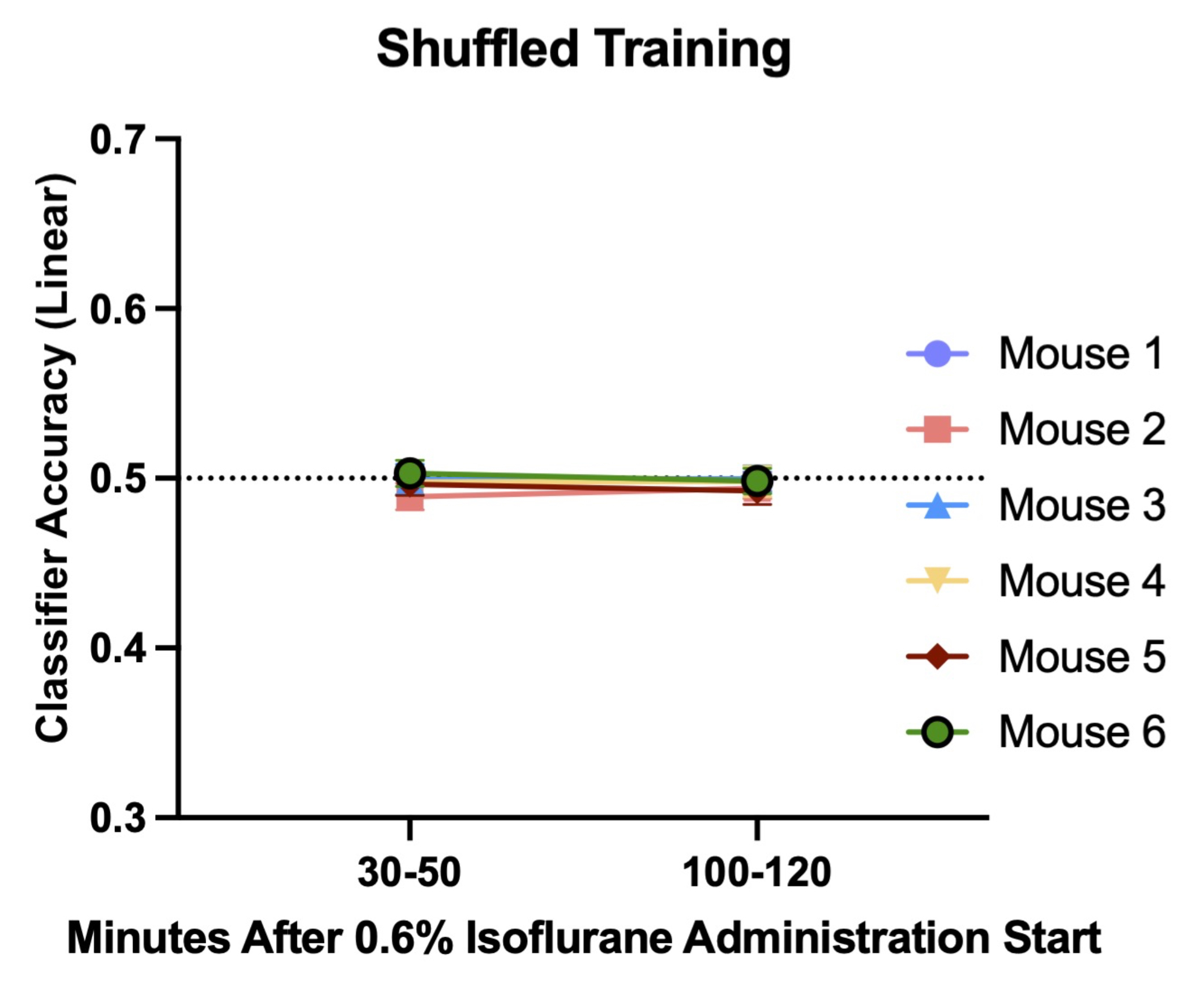

Supplement: Supplementary Figure 1 — Classifier accuracy built on shuffled training data does not trend from chance over time. The accuracy of linear classifiers built from shuffled training data do not trend in any direction over time (p = 0.4288, 2-way ANOVA), but rather remain at the chance. This demonstrates a lack of inherent directional bias of the model itself over time, suggesting trends over time seen with non-shuffled training data are real. [file Image_1.JPEG]
